# Poly(Lactic Acid) Block Copolymers with Poly(Hexylene Succinate) as Microparticles for Long-Acting Injectables of Risperidone Drug

**DOI:** 10.3390/polym14194111

**Published:** 2022-09-30

**Authors:** Iouliana Chrysafi, Stavroula Nanaki, Alexandra Zamboulis, Margaritis Kostoglou, Eleni Pavlidou, Dimitrios N. Bikiaris

**Affiliations:** 1 Laboratory of Advanced Materials and Devices, Department of Physics, Faculty of Sciences, Aristotle University of Thessaloniki, GR-541 24 Thessaloniki, Greece; 2 Laboratory of Polymers Chemistry and Technology, Department of Chemistry, Faculty of Sciences, Aristotle University of Thessaloniki, GR-541 24 Thessaloniki, Greece; 3 Laboratory of Chemical and Environmental Technology, Aristotle University of Thessaloniki, GR-541 24 Thessaloniki, Greece

**Keywords:** poly(lactic acid), poly(hexylene succinate), Risperidone, long-acting injectable, drug delivery, enzymatic hydrolysis, drug release

## Abstract

In the present work, Risperidone microparticles from poly(lactic acid)/poly(hexylene succinate) (PLA-b-PHSu) block copolymers in different ratios, 95/05, 90/10 and 80/20 *w*/*w*, were examined as long-acting injectable formulations. Nuclear magnetic resonance (NMR) was used to verify the successful synthesis of copolymers. Enzymatic hydrolysis showed an increase in weight loss as the content of PHSu increased, while the cytotoxicity studies confirmed the biocompatibility of the copolymers. The polyesters were further used to encapsulate Risperidone by spray drying. The drug-loaded microparticles were studied by Fourier transform infrared spectroscopy (FTIR), scanning electron microscopy (SEM) and X-ray diffraction (XRD). SEM microphotographs confirmed that spherically shaped microparticles were prepared with sizes about 5–12 μm, while XRD and differential scanning calorimetry (DSC) studies evidenced that Risperidone was encapsulated in amorphous form. The drug loading and the entrapment efficiency of Risperidone were studied as well as the in vitro release from the prepared microparticles. As the content of PHSu increased, a higher release of Risperidone was observed, with PLA-b-PHSu 80/20 *w*/*w* succeeding to release 100% of RIS within 12 days. According to theoretical modeling, the kinetics of RIS release from PLA-b-PHSu microparticles is complex, governed by both diffusion and polymer erosion.

## 1. Introduction

Aliphatic polyesters are gaining more and more ground in research and, therefore, in industry, due to their biodegradability. They can be characterized as biodegradable because their repeated units are bonded via ester linkages, which are enzymatically degradable since they are found in nature in a wide variety [1]. Aliphatic polyesters can be used in a broad range of applications. They are mostly known for food packaging, but due to their biocompatibility, they can also be used in tissue engineering, as artificial implants, or as matrices for drug delivery systems. Some of the most famous aliphatic polyesters are poly(lactic acid) (PLA), poly(glycolic acid) (PGA) and their copolymers (PLGA), polycaprolactone (PCL), poly(hydroxybutyrate) (PHB), poly(butylene succinate) (PBSu), poly(ethylene succinate) (PESu), etc. [2]. Nearly all their monomers can be produced from renewable resources and, thus, can replace petroleum-based plastics.

Poly(lactic acid), PLA, is currently used in numerous applications, such as in the food industry, in textiles and in medicine. Specifically, some biomedical applications include orthopedic and fixation devices, tissue engineering, wound healing and drug delivery systems [3,4]. Low-molecular-weight PLA derives from the direct polymerization of lactic acid through heating, while high-molecular-weight PLA can be synthesized by ring-opening polymerization [5]. Lactic acid can be produced by chemical processes or by fermentation. In the latter case, several substrates can be used as carbohydrate sources, such as whey, barley, sugarcane, soybean, milk, corn, sulfite waste liquor and potatoes, and by selecting the fermentation bacteria, the L, the D or both isomers are produced.

Although in the literature, PLA is considered as a biodegradable polymer [5], nowadays, its degradability is disputed. The degradation process of PLA has been thoroughly studied in compost, soil environments and by hydrolytic, enzymatic, photodegradative, microbial and thermal degradation [6,7,8,9]. Most research showed that PLA is biodegradable only in industrial composting conditions [10], while, when considering the definition of biodegradability (a material that can degrade to carbon dioxide and water by microbial action under normal environmental conditions [8]), the effect of the degradation mechanisms mentioned above could incorrectly be supposed as natural reactions. It cannot be ignored that the degradation time of other conventional plastics, such as poly(ethylene) and polystyrene, that fluctuate from 500 to 10,000 years, are, by far, higher compared to PLA, which takes about 6 months to 2 years to degrade. The degradation of PLA depends on different factors, such as molecular weight, crystallinity, purity, temperature, pH, presence of terminal carboxyl or hydroxyl groups, water permeability, and additives that act catalytically and may include enzymes, bacteria or inorganic fillers [11]. Concerning PLA in medicine, it is not only used due to its ability to degrade in humans’ body but also due to its biocompatibility. The degradation process is carried out by microorganisms when PLA is excreted from the body, without causing undesirable effects [12]. The main degradation mechanism of PLA inside the body includes the hydrolysis of the ester-bond backbone. Lactic acid oligomers or monomers are produced during degradation of the inside bulk polymer and its surface. Subsequently, new carboxylic groups at the terminal ends of the cleaved PLA chains are formed and lead to the catalysis of the hydrolytic degradation. Moreover, internal cavities are formed due to the degradation caused by the diffusion of water into the polymer bulk. The hydrolyzing water molecules will diffuse first, while the cleaved monomers will take more time to diffuse out of PLA [13].

PLA seems to exhibit a low-enzymatic hydrolysis, both in vitro and in vivo [14]. Although suitable for some long-term applications, this feature limits the use of neat PLA in other biomedical applications. Thus, modification by surface treatment, stereocomplexation, copolymerization and blending with other polymers or nanoadditives has been used to modulate its biodegradability [15,16,17]. To successfully synthesize a PLA-based material for drug delivery applications, the relatively negligible biodegradation of PLA, resulting in a slow and uncontrolled release of the encapsulated drug, should be tackled. The slow release of the encapsulated drug is attributed to the hydrophobic interactions and the slow hydrolysis of the ester linkages of the PLA backbone that cause delayed diffusion through the PLA core [18].

When designing drug delivery systems, two important aspects must be taken into account: the pharmaceutical form (tablets, creams, patches, injectable solution, nanoparticles, microparticles, etc.) and the route of administration (oral, parenteral, pulmonary, transdermal, intranasal, cardiovascular, etc.) [19]. Most controlled systems today aim at treating chronic diseases, such as cancer, neurodegenerative diseases and infectious diseases [20], and the abovementioned aspects adapt to the specific needs of each disease. Schizophrenia is a neuropsychiatric chronic disease that affects around 1% of the population, mainly adolescents and young adults, and can be characterized by episodes in which the patient cannot distinguish between real and unreal experiences. Risperidone (RIS) belongs to the first generation of antipsychotic drugs that is an effective treatment for those patients. The daily administration of RIS in the form of pills or tablets has shown positive results in patients’ mental health. Despite that, it has been observed that patients, after a certain time period, believing that they have been cured, discontinue their treatment and do not comply with the instructions of the medical staff and relatives, resulting in the patient’s regression. Thus, it was deemed necessary to find a formulation that would have the same results, be easier to administer and give a sense of non-illness to patients. The solution was provided by long-acting injectables (LAIs), the second generation of antipsychotics [21,22].

LAIs are drug delivery systems that are administered topically, by intramuscular administration, creating a “depot” of drug, which is released at a controlled rate and leads to the creation of a specific concentration of the drug in the human body. The advantages over other formulas are the avoidance of unnecessary drug concentrations in the liver and the always “available” amount of drug in the human body. Concerning the patient as a person, they reduce the risk of overdose and regular appointments with the doctor ensure the effective control of the patient’s progress [23]. However, the reduced flexibility of administration and the prolonged period that the organism has to adapt to the dose and to achieve a steady state are some of the LAIs’ disadvantages [22]. Specifically, as far as the commercial form of Risperidone is concerned, the period of 7–8 days until the start of its release, due to the slow hydrolysis of poly(lactic-co-glycolic acid) (PLGA), is a major disadvantage. Therefore, during the first week of administration, co-administration of Risperidone tablets is required to achieve the desired concentration of the drug [24].

Microparticles or microcapsules have been developed as drug carriers in controlled systems, due to their advantages. Mainly, microencapsulation offers prolonged and controlled release of the active substance, prevention from the interaction between drugs, targeted and more effective action, etc. [25,26]. There are several techniques to encapsulate an active substance for drug delivery systems, which can be divided into two main categories. In the first category, the starting materials are monomers or prepolymers, while chemical processes are taking place during microsphere formation. In the second category, polymers are the starting materials and, thus, no chemical reactions are involved, while the process simply entails the shape production [25]. The most used microencapsulation techniques are those of spay drying, emulsion evaporation or diffusion method and salting out method. In the spray-drying method, the liquid feed is first atomized and then pumped into a hot-drying chamber, where droplets solidify into particles that are collected at the end [26]. In the emulsion evaporation method, the drug is dissolved at first in an organic solvent containing the polymer and then the emulsification of the organic phase (dispersed phase) in an aqueous phase (continuous phase) takes place. Subsequently, the continuous phase extracts the solvent from the dispersed phase, followed by solvent evaporation and transformation of the droplets into solid particles. At the end, the microspheres are recovered and dried to remove any remaining solvent [27]. During the salting out method, the polymer and the drug are dissolved into the organic solution and then are emulsified in an aqueous phase under stirring. Finally, water is added to diffuse acetone into the water and to precipitate the polymer in the form of microparticles [28].

In the present study, new poly(lactic acid)/poly(hexylene succinate) (PLA-b-PHSu) block copolymers were studied in different PLA/PHSu ratios as matrices for the encapsulation and delivery of Risperidone. The synthesis and part of the characterization of the copolymers have already been published in a previous article [29] and the same samples were used in the current study for the microencapsulation. It was considered rather important to thoroughly examine all the properties of the materials, notably their biocompatibility and degradability, to determine their suitability as drug delivery matrices. PHSu is a linear polyester, which was chosen to form copolymers with PLA, due to the non-toxicity, biodegradability and biocompatibility of succinic acid [3,29]. Risperidone-loaded microspheres of PLA-b-PHSu were further prepared and the extended and controlled release from the PLA-b-PHSu formulations was studied for the first time. The spray-drying method was used to prepare proper microparticles because it is a relatively simple continuous process and faster, more reproducible and scalable method compared to others [26]. Moreover, it is easy to control the features of the particles and their properties can be kept constant during the ongoing process. Finally, an attempt to quantify the drug release kinetics was performed to gain further insights in the release mechanism.

## 2. Materials and Methods

### 2.1. Materials

L-lactide (98+%) was obtained by Alfa Aesar Chemicals (Kandel, Germany). Succinic acid (ACS reagent, ≥99.0%), 1,6-hexanediol (99%), tetrabutyl titanate (TBT) (97%) and tin(II) 2-ethylhexanoate (TEH) (96%), were purchased by Sigma-Aldrich chemical company (Saint Louis, MO, USA). Rhizopus delemar and Pseudomonas cepacia lipases were purchased from BioChemika. Risperidone API (RIS) was kindly donated by Pharmathen S.A. All other chemicals and reagents were of analytical grade.

### 2.2. Synthesis of PLA/PHSu Copolymers

PLA-b-PHSu copolymers in 95/5, 90/10 and 80/20 mass ratios were synthesized by a combination of melt polycondensation and ring opening polymerization (ROP) as described in our previous work [29]. Briefly low-molecular-weight PHSu was synthesized by two-stage melt polycondensation, using TBT as catalyst, and an average number molecular weight (Mn) of about 5000 g/mol was obtained. In the second stage, low-molecular-weight PHSu was used to synthesize the block copolymers by ROP of L-lactide in the presence of TEH as catalyst. By size exclusion chromatography (SEC) it was found that the prepared PLA-b-PHSu 95/5, 90/10 and 80/20 *w*/*w* copolymers have Mn values of 44,000, 33,000 and 21,700 g/mol, respectively, while the Mn of neat synthesized PLA was 47,000 g/mol [29].

### 2.3. Characterization Methods

#### 2.3.1. Nuclear Magnetic Resonance (^1^H-NMR, ^13^C-NMR)

The chemical structure and composition of the synthesized copolymers were characterized by ^1^H and ^13^C NMR spectroscopy. NMR spectra were recorded on a spectrometer (Agilent AM 600, Agilent Technologies, Santa Clara, CA, USA) operating at a frequency of 600 MHz for protons at room temperature. Deuterated chloroform (CDCl_3_) was used as solvent to prepare polymer solutions. The spectra were calibrated using the residual solvent peak. Thus, 32 and 512 scans were recorded for the ^1^H and ^13^C spectra, respectively.

#### 2.3.2. Enzymatic Hydrolysis

For the evaluation of the degradation process, films of each sample (PLA, PLA-b-PHSu95/05, PLA-b-PHSu90/10 and PLA-b-PHSu80/20) were prepared with a PW 30 Otto Weber (Dusseldorf, Germany) hydraulic press connected to a temperature controller (Omron E5AX, Dusseldorf, Germany). The samples were heated until their melting temperature and the films formed were cut in 3 cm × 3 cm squares with an approximate thickness of 0.3 mm. The samples were incubated at 37 ± 1 °C for 40 days in suitable Petri dishes containing phosphate-buffered saline (PBS) (pH = 7.4) with Rhizopus delemar and Pseudomonas cepacia lipases at 0.09 and 0.01 mg/mL content, respectively. After specific time intervals, the films were removed from the Petri dishes, washed twice with distilled water and dried in vacuum at 30 °C. After 24 h the samples were weighed, and the procedure repeated until a constant weight was achieved. The biodegradation degree was estimated from the weight loss of the weighed polymer according to the following equation.

(1) $$\mathrm{Weight}\;\mathrm{loss}\;\left(\%\right)=\frac{w_{0}-w_{r}}{w_{0}}\cdot 100\%$$

where w_0_ is the weight of the films before degradation and w_r_ is the weight of the films after degradation and drying.

#### 2.3.3. Scanning Electron Microscopy (SEM)

Scanning electron microscopy was used to investigate the morphology of PLA and its copolymers after enzymatic hydrolysis. A second film was prepared and a small piece from each sample was obtained before and after the last hydrolysis period interval. The structure of synthesized microparticles and Risperidone were also observed by SEM, as well as the inner surface of the microparticles. In order to study the internal surface, PLA and its copolymers were mixed with an epoxy resin, suitable for electron microscopy specimens. Then, the resulting slurry compounds were molded into small caps to form tablets and left to harden overnight. After hardening, the tablets were grinded and polished to mirror finish by various-grained diamond lapping films using an Allied MultiPrep automated polisher. The cross-sectional surface of RIS microparticles was observed. The morphology of the Risperidone microspheres was also studied after the dissolution experiments. The films as well as the microparticles and Risperidone were placed on aluminum stubs, settled with silver paint and finally carbon coated. Microphotographs were taken from the surface area with a scanning electron microscope (Jeol microscope—J.S.M. 7610F, Tokyo, Japan).

#### 2.3.4. In Vitro Cytotoxicity Study

Τhe cytotoxicity of PLA, PHSu and their copolymers was evaluated by measuring the viability of human skin fibroblasts. Cell viability was determined by the “3-(4,5-dimethylthiazol-2-yl)-2,5-diphenyl tetrazolium bromide” (MTT) assay. The samples were seeded in 96-well plates at a density of 5000 cells per well. Each bulk sample in the form of films (PLA, PHSu, PLA-b-PHSu copolymers) was cut into 3 equal pieces and distributed into 3 wells to have 3 replicates to increase reliability. Twenty-four hours after seeding, the samples were added in the wells and after 24 h of incubation at 37 °C, 50 μL of MTT solution (5 mg/mL in PBS pH 7.4) was added into each well and plates were incubated at 37 °C for 4 h. The medium was withdrawn and 50 μL of DMSO was added to each well and agitated thoroughly to dissolve the formed crystals. Then, the supernatants were transferred to a new plate to measure the optical density (by spectrophotometer (PerkinElmer, Waltham, MA, USA), at 570/630 nm). The samples were also studied after 72 h of incubation as positive controls of proliferation of equal numbers of human skin fibroblasts were seeded onto plastic and assayed similarly. The results are expressed as mean percentage viability (±SEM) compared with untreated controls (analyzed by ANOVA).

(2) $$\mathrm{Cell}\;\mathrm{viability}=\frac{\mathrm{number}\;\mathrm{of}\;\mathrm{cells}\;\mathrm{treated}\;\mathrm{with}\;\mathrm{the}\;\mathrm{nanoparticles}\;}{\mathrm{non}-\mathrm{treated}\;\mathrm{cells}\;\left(\mathrm{control}\right)}\cdot 100$$

### 2.4. Preparation and Characterization of Risperidone Microparticles

#### 2.4.1. Preparation of Risperidone Microparticles

Microparticles were prepared by spray drying with a Mini Spray-Dryer B-290 (BUCHI Labortechnik AG, Flawil, Switzerland). The solutions for spray drying were obtained by completely dissolving 5 g of each polymer and 0.85 g of Risperidone in 200 mL of dichloromethane (DCM). A 2.2 μm nozzle tip was used while spraying conditions were: inlet temperature 75 °C, outlet temperature 65 °C, aspirator 100%, Q-flow at 30 and pump at 40%. The collected microparticles were stored under vacuum until further use. Neat microparticles of PLA and PLA-b-PHSu90/10 were also prepared for comparison.

#### 2.4.2. Fourier Transform Infrared Spectroscopy (FTIR)

FTIR measurements were performed with a Cary 670 spectroscope from Agilent Technologies (Palo Alto, CA, USA) equipped with a diamond-attenuated total reflectance (ATR) accessory (GladiATR, Pike Technologies, Madison, WI, USA). A small piece of each sample was placed in the ATR apparatus without any further preparation and the spectra were collected in the mid IR area (4000–400 cm^−1^), with 32 scans and a resolution of 4 cm^−1^.

#### 2.4.3. X-ray Diffraction (XRD)

A two-cycle Rigaku Ultima + powder X-ray diffractometer with CuKa radiation was used to study the structure and the degree of crystallinity of the samples. Diffraction spectra were obtained over a 2θ range of 5–60° with a step size 0.05° and step time 1.5 s, operating at 40,000 V and 0.03 A. The characterized peaks of each sample were identified by the Joint Committee on Powder Diffraction Standards (JCPDS) database [30] and according to literature. PLA and its copolymers were measured as thin films placed in a specific sample holder, while PHSu, Risperidone and the microparticles were measured as powders.

#### 2.4.4. Differential Scanning Calorimetry (DSC)

The thermal behavior of Risperidone and the prepared microspheres was tested using a differential scanning calorimeter (DSC) from Netzsch equipped with Intracooler (DSC Polyma 214). The measurements of Risperidone and the microparticles were obtained during the first heating from 20 °C to 200 °C, at a rate of 10 °C/min under nitrogen atmosphere. Samples were weighted (about 5 mg) by a Kern analytical balance and sealed in aluminum pans.

#### 2.4.5. Thermogravimetric Analysis (TGA)

A SETARAM SETSYS TG-DTA 16/18 was used for the thermogravimetric analysis of Risperidone and the microparticles. The samples (5 ± 0.5 mg) were placed in alumina crucibles and heated from 20 °C to 200 °C, at a rate of 10 °C/min, in a 50 mL min^−1^ flow of N_2_. Continuous recordings of sample temperature, sample mass, its first derivative and heat flow were taken.

#### 2.4.6. In Vitro Drug Release

For the in vitro release studies, a Distek Dissolution Apparatus (Evolution 2100C, North Brunswick Township, NJ, USA) was used, equipped with an autosampler (Evolution 4300, North Brunswick Township, NJ, USA), using the basket method (United States Pharmacopeia, USP I method). Microspheres, placed into suitable dialysis tubing cellulose, were inserted into the dissolution baskets, while the dissolution analysis was performed at 37 ± 1 °C with a rotation speed of 50 rpm. The dissolution medium was 1000 mL of a phosphate-buffered saline (PBS), pH = 7.4 solution. At predefined time intervals, 2 mL of aqueous solution was withdrawn from the release media and analyzed for Risperidone content by HPLC (the method is described below). The drug loading and the drug entrapment efficiency of microspheres were calculated according to the following equations:

(3) $$\mathrm{Drug}\;\mathrm{loading}\;\left(\%\right)=\frac{\mathrm{weight}\;\mathrm{of}\;\mathrm{drug}\;\mathrm{in}\;\mathrm{microparticles}\;}{\mathrm{weight}\;\mathrm{of}\;\mathrm{microparticles}}\cdot 100$$

(4) $$\mathrm{Entrapment}\;\mathrm{efficiency}\;\left(\%\right)=\frac{\mathrm{weight}\;\mathrm{of}\;\mathrm{drug}\;\mathrm{in}\;\mathrm{microparticles}}{\mathrm{weight}\;\mathrm{of}\;\mathrm{initially}\;\mathrm{used}\;\mathrm{drug}}\cdot 100$$

#### 2.4.7. HPLC Method

Risperidone content was assayed using a well-established HPLC method previously used in our lab [31]. In brief a Shimadzu Prominence HPLC system consisting of a degasser (Model DGU-20A5, Tokyo, Japan), a pump (Model LC-20AD, Tokyo, Japan), an autosampler (Model SIL-20AC, Tokyo, Japan), a UV-Vis detector (Model SPD-20A, Tokyo, Japan) and a column oven (Model CTO-20AC, Tokyo, Japan) was used. Chromatographic analysis was performed with a C18 column (CNW Technologies Athena, 120 Å, 5 μm, 250 mm × 4.6 mm, Tokyo, Japan) at 25 °C. Methanol/H_2_O/triethylamine at a ratio of 80/19.5/0.5 *v*/*v*/*v* was used as the mobile phase, while acetic acid was used to adjust the pH at 10.2. The flow rate was set at 1.0 mL/min, injection volume was 20 μL, while the Risperidone analysis was performed at 254 nm.

## 3. Results and Discussion

### 3.1. Characterization Methods of the Synthesized Copolymers

#### 3.1.1. Nuclear Magnetic Resonance (^1^H-NMR, ^13^C-NMR)

**Figure 1:**
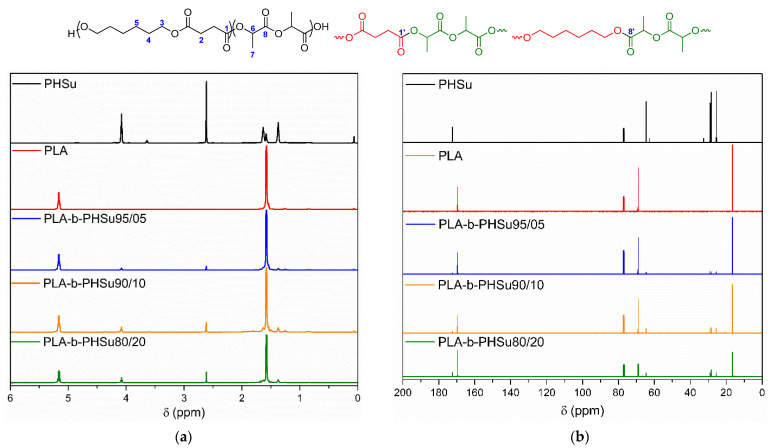
^1^H (**a**) and ^13^C NMR (**b**) spectra of PLA, PHSu and their copolymers.

PLA, PHSu and its PLA-b-PHSu copolymers were characterized by NMR and the recorded spectra are presented in Figure 1. NMR spectra confirmed the successful preparation of the copolymers and, furthermore, suggest a block structure. In the PHSu ^1^H NMR spectrum, the peak at 1.36 pm is assigned to the four central methylene protons of 1,6-hexanediol (-CH_2_- 5), while the peaks at δ = 1.60 ppm are assigned to methylene protons 4 [32]. The resonance signal at 2.60 ppm corresponds to the methylene protons of succinic acid (C(O)CH_2_ 2) [33]. Finally, the resonance signal at 4.06 ppm corresponds to the -OCH_2_- methylene protons of HD (CH_2_ 3), while the peak at 3.63 ppm is attributed to end-chain CH_2_OH groups [34]. As for PLA, two main peaks can be observed at δ = 1.58 and 5.15 ppm that are attributed to the methyl proton (CH_3_ 7) and methine groups (CH 6) of PLA, respectively [35,36]. The spectra of the copolymers exhibit both signals attributed to PHSu and PLA and the peaks corresponding to PHSu units (1.36, 2.60 and 4.06 ppm) increase proportionally to the PHSu content (vide infra).

The following attribution was made for the ^13^C spectrum. Peaks at 16.6 and 69.0 ppm are attributed to the carbon atoms of the CH_3_ 7 and CH 6 groups of PLA, while the peak at δ = 169.5 ppm corresponds to the carbonyl of the ester groups C(O) 8. In the PHSu ^13^C NMR spectrum, peaks at 25.5, 28.4 and 64.6 are attributed to the methylene groups of HD: CH_2_ 5, CH_2_ 4 and CH_2_ 3, respectively. Finally, the resonance signal at 172.4 ppm is assigned to the carbonyl moiety of the ester groups C(O) 1. In the copolymer ^13^C spectra, a similar trend to the ^1^H spectra is observed.

The composition of the final copolymers was calculated by comparing the integrations of CH_2_ 3 and CH 6. The percentages calculated by NMR were in total agreement with the feed molar ratio (the calculated PHSu percentages were: 18% (PLA-b-PHSu80/20), 11% (PLA-b-PHSu90/10) and 5% (PLA-b-PHSu95/5). The blocky architecture of the copolymers was estimated based on the resonance signals at 170.1 and 172.2 ppm, which are attributed to lactide units linking to hexanediol units (C(O) 8′) and succinic units linking to lactide units (C(O) 1′), respectively. Indeed, these peaks are very small compared to peaks at 169.5 and 172.4 ppm, indicating that there are very few lactide/succinic or lactide/hexanediol junctions, thus, suggesting block copolymers.

#### 3.1.2. Enzymatic Hydrolysis

A biomaterial’s degradation mainly originates from four causes: hydrolytic, enzymatic, oxidative and physical degradation. When the bond scission is catalyzed by enzymes, the process is referred to as enzymatic degradation. The process of enzymatic degradation can theoretically be divided into four individual steps. Firstly, the enzymes diffuse on the substrate interface (1), followed by absorption and complexation (2). Then, the catalysis of the scission reaction actually takes place (3) and, finally, the soluble degradation products diffuse away from the substrate surface (4) [10]. Due to their large size, extracellular enzymes cannot penetrate deep into the polymeric matrix and act only on the polymer surface; in this case, biodegradation essentially concerns only surface erosion [1]. In addition to diffusion, the erosion of the polymeric matrix, through hydrolysis of the ester groups, affects drug release in polyester-based drug delivery systems. Therefore, the evaluation of hydrolysis is considered crucial.

**Figure 2:**
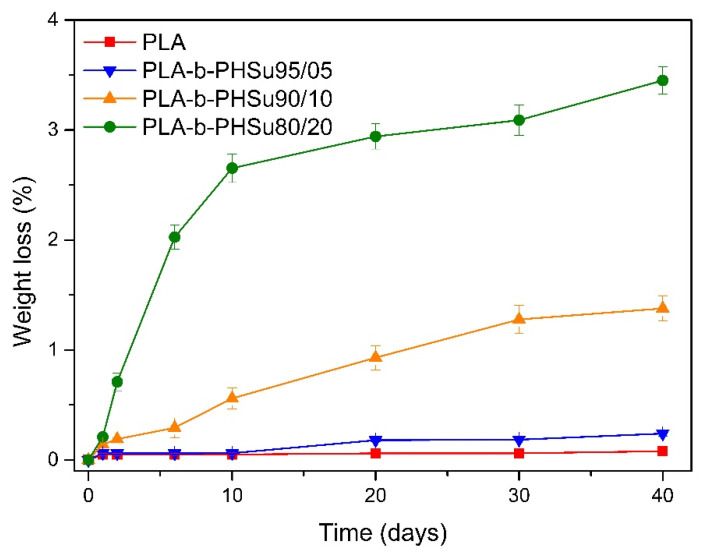
Weight loss versus time of neat PLA and PLA-b-PHSu copolymers during enzymatic hydrolysis test.

The enzymatic hydrolysis of the copolymers was studied in the presence of Rhizopus delemar and Pseudomonas cepacia lipases. Since lipases are able to be activated by adsorption on hydrophobic surfaces, they can cleave ester bonds in the solid phase. From Figure 2, neat PLA and PLA-b-PHSu95/05 *w*/*w* showed negligible weight loss during hydrolysis. Similarly, PLA-b-PHSu90/10 *w*/*w* also exhibited a limited weight loss: approximately 1.2% after 30 days of incubation in the enzyme solution. Extremely low hydrolysis of PLA is also observed in the literature [37] and is attributed to the hydrophobic nature of PLA in combination with the rather low temperature used (37 °C), compared to the higher glass transition and melting temperatures (Tg ≈ 50 °C, Tm ≈ 140 °C) [29]. Beslikas [14] in order to accelerate degradation, studied enzymatic hydrolysis at 50 °C, where he achieved ca. 3.5% weight loss after 60 days, a higher value compared to the present study (ca. 0.1% after 40 days). PLA-b-PHSu80/20 *w*/*w* showed the highest weight loss: 3.5% loss of the initial mass after 40 days of enzymatic hydrolysis. Based on the above results, it can be concluded that polymer erosion through enzymatic hydrolysis may be manipulated by adjusting the content of PHSu in the copolymers.

#### 3.1.3. Surface Examination of Copolymers by Scanning Electron Microscopy

For a thorough study of the effect of enzymatic hydrolysis, it is rather important to know how the hydrolysis proceeds within the polymer matrices, except from the weight loss that provides a general trend of the rate of enzymatic hydrolysis. Thus, in order to examine the morphology of the polymeric films and the hydrolysis process, scanning electron microscopy (SEM) experiments were performed. Different pieces of each sample were studied before and after enzymatic hydrolysis. Figure S1 (Supplementary Materials) shows the microphotographs taken before hydrolysis on the left and after 40 days of hydrolysis on the right, for PLA and its copolymers. Generally, it can be concluded that the surface of the films becomes slightly rougher with hydrolysis and some holes appear, while initially, the surface of the samples was quite smooth. Specifically, it can be observed that neat PLA displays no significant differences, which was expected in accordance with the weight loss. The surface of the PLA-b-PHSu95/05 sample after hydrolysis is also quite smooth, while PLA-b-PHSu90/10 presents increased roughness. Most differences show up in the PLA-b-PHSu80/20 sample after hydrolysis (Figure S1h). Inhomogeneous patterns are visible on the entire surface of the sample, especially in the lighter regions, corresponding to the polymer erosion that is affected by enzymatic hydrolysis. The results are in total agreement with the study of the enzymatic hydrolysis as PLA-b-PHSu80/20 presented the highest weight loss.

#### 3.1.4. Cytotoxicity Study

**Figure 3:**
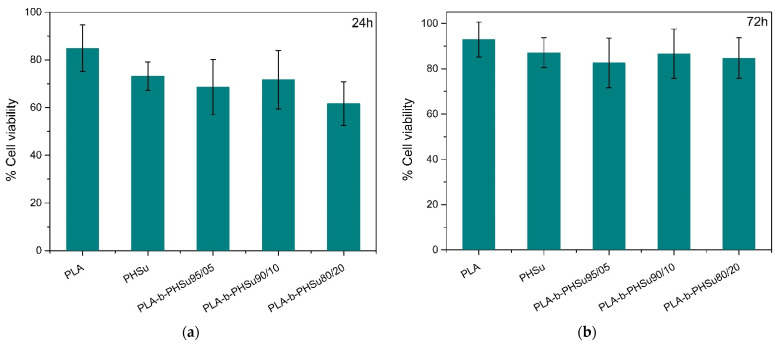
Cell viability of neat PLA, PHSu and PLA-b-PHSu copolymers after 24 (**a**) and 72 (**b**) h. Results are percentage values (Mean ± SEM) where 100% corresponds to control values (untreated cells). No significant differences between groups are indicated by one-way ANOVA.

PLA is extensively used in drug delivery systems due to its low cytotoxicity. Succinic acid has also been used in different applications, such as in treating lung cancer [38], for hepatic encephalopathy [39] and for bone repair [40], etc. However, poly(hexylene succinate) has never been used in drug delivery systems; thus, it is crucial to examine the cytotoxicity of PHSu and its PLA-b-PHSu copolymers. Figure 3 shows the cells’ viability after 24 and 72 h of incubation. Although cell viability was under 80% for most of the samples after 24 h, this is common to many studies. As seen in Figure 3b, the cell viability of the samples increases, reaching 90% for PLA. Therefore, it can be assumed that the cells can prove resistant to an initially cytotoxic stimuli by increasing their proliferation after 24 h of culture. Considering those results, it is clear that the copolymers are non-toxic; thus, they are biocompatible and can be adopted as drug delivery systems. One-way ANOVA statistical tests were applied in order to study the correlation between our samples. In the case of the results at 24 h, the *p* value was 0.67, while at 72 h, *p* was 0.63. Therefore, *p* is higher than 0.05 and, thus, there are no significant differences between the samples.

### 3.2. Microparticle Preparation and Study

#### 3.2.1. Scanning Electron Microscopy (SEM)

**Figure 4:**
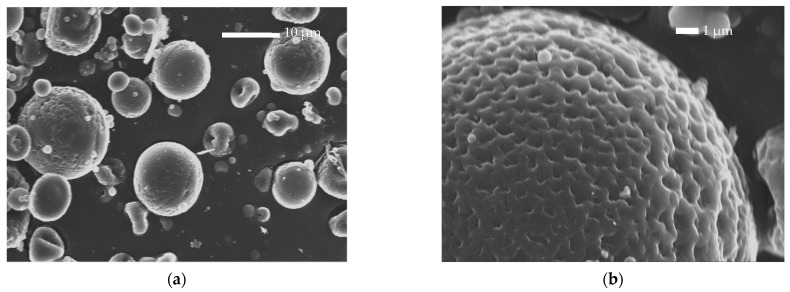

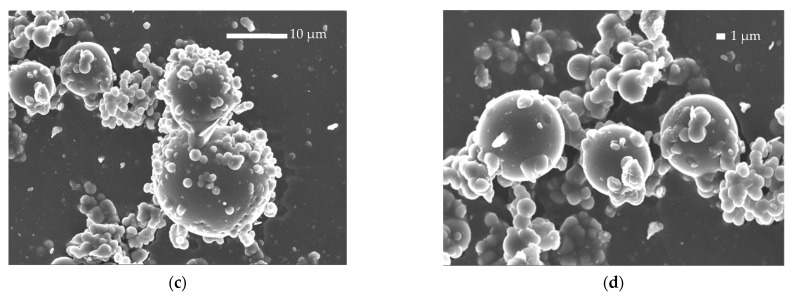
SEM microphotographs of neat PLA microparticles (**a**,**b**) and neat PLA-b-PHSu 90/10 microparticles (**c**,**d**).

**Figure 5:**
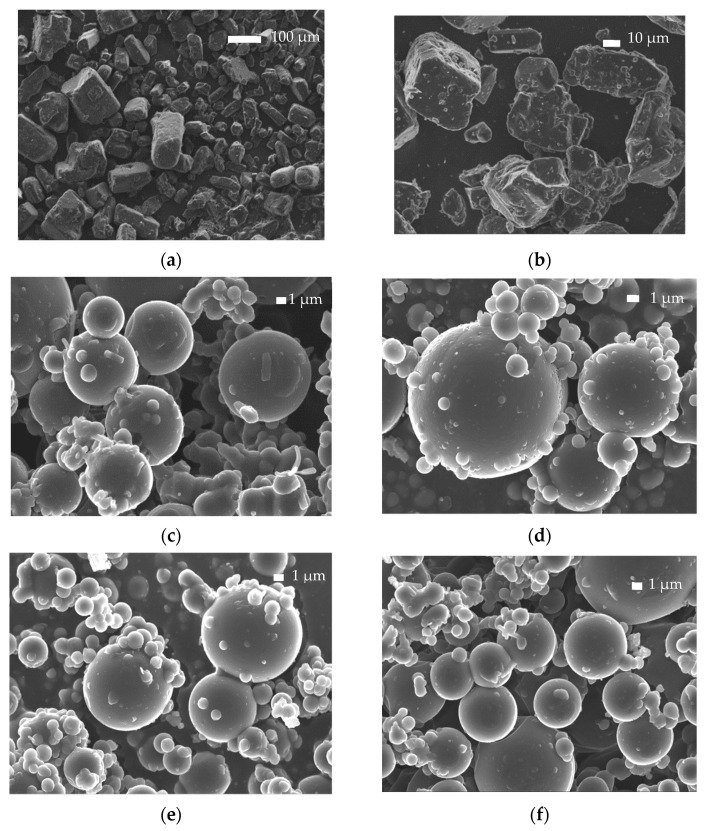
SEM microphotographs of Risperidone API (**a**,**b**) and PLA-b-PHSu microparticles containing Risperidone in 100/0 (**c**), 95/05 (**d**), 90/10 (**e**), 80/20 (**f**) ratios.

The studies performed on bulk PLA-b-PHSu copolymers confirmed their suitability for drug delivery applications. Consequently, microparticles with (PLA and all copolymers) and without drug (PLA and PLA-b-PHSu90/10 only) were prepared by spray drying. As can be observed by the SEM photos (Figure 4 and Figure 5), spherical microparticles were successfully obtained.

Neat PLA microparticles exhibited sizes ranging between 8 and 12 μm, while those prepared by neat PLA-b-PHSu90/10 *w*/*w* had sizes of 6–10 μm (Figure 4). Smaller particles that seem to be deposited on their surface are also observed and especially in those prepared by a copolymer with higher PHSu ratio. This is probably due to the temperature used for the spray-drying procedure. Indeed, the apparatus temperature was set at 75 °C, resulting in 60 °C right before the entrance of the vessel. This temperature is higher than the Tg values of the synthesized copolymers (between 30 and 52 °C and increasing with increasing PLA content [29]). It was reported that, when spray drying is performed at higher temperatures than the Tg value of PLA, agglomeration and adhesion of microparticles were observed [41,42].

Figure 5a,b show the SEM photos of neat Risperidone. As can be observed, Risperidone is a crystalline powder, orthorhombic in shape, with particle sizes between 30 and 100 μm. Figure 5c–f also show the Risperidone-containing microparticles prepared by spray drying. It can be observed that spherical microparticles were successfully prepared with sizes varying between 5 and 12 μm. Analogous observations to the empty microparticles were also made for Risperidone-loaded microparticles. In all formulations, adhesion of smaller microspheres to the surface of the bigger ones was observed due to the temperature used during the spray-drying process, as referred to previously.

Microspheres prepared using PLA showed pores on their surface attributed to the rapid evaporation of the solvent. Surface pore formation is also observed at lower extent in the microparticles prepared by PLA-b-PHSu copolymers. Another observation that must be considered is that in 90/10 and 80/20 ratios, there are black spherical shadows inside microparticles. Their morphology seems to be like those formed in poly-nuclear microcapsules [43] with the difference being that no drug is expected to be entrapped inside.

**Figure 6:**
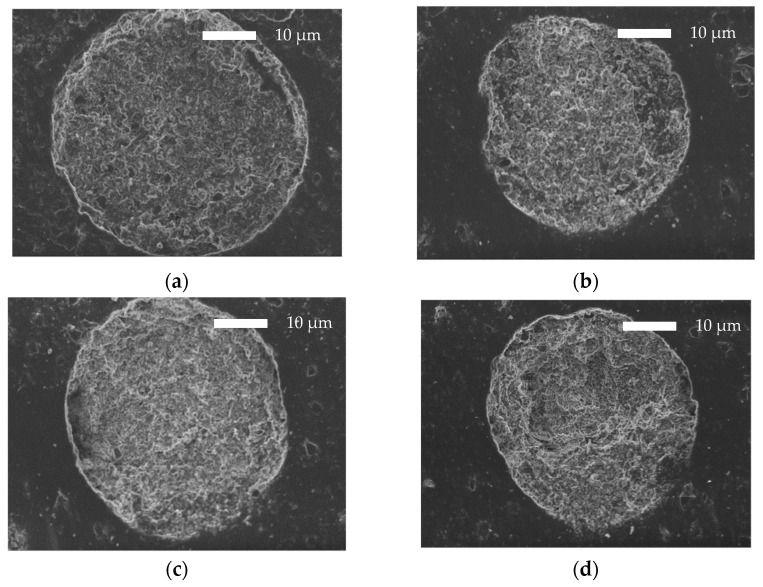
SEM microphotographs of the cross-sectional surface of PLA-b-PHSu microparticles containing Risperidone in 100/0 (**a**), 95/05 (**b**), 90/10 (**c**), 80/20 (**d**) ratios.

In Figure 6, the cross-sectional morphology of RIS microspheres can be observed. The microphotographs present quite the same morphology in each sample. A thin layer of the polyester surrounding the microparticles can be seen. The cracks on the copolymers are attributed to the polishing procedure.

#### 3.2.2. Fourier Transform Infrared Spectroscopy

**Figure 7:**
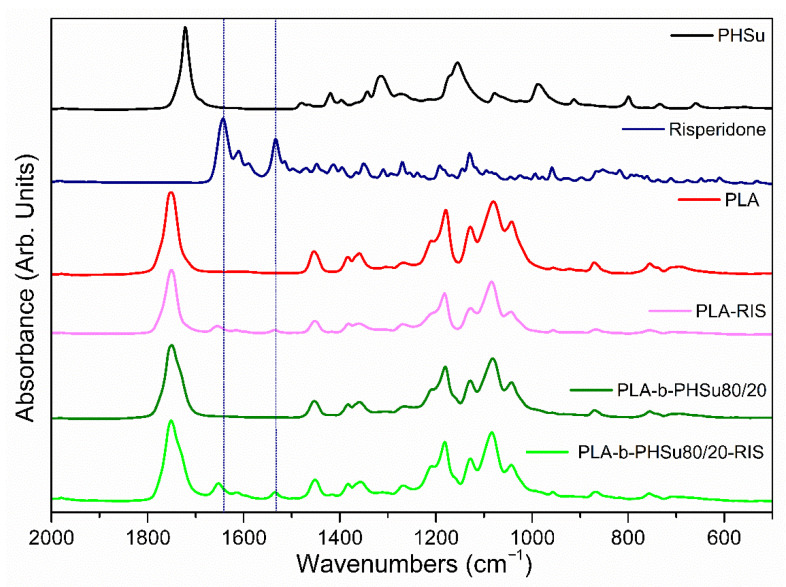
FTIR spectra of PHSu, Risperidone, PLA, PLA-bPhsu80/20 and of the PLA-RIS and PLA-b-Phsu80/20-RIS prepared microparticles.

FTIR spectroscopy was used to detect the effect of Risperidone in the microparticles. Figure 7 shows the FTIR-ATR spectra of neat PHSu, Risperidone, PLA and PLA-b-PHSu 80/20 *w*/*w* as well as the Risperidone microparticle spectra. PLA-b-PHSu 95/05 *w*/*w* and PLA-b-PHSu 90/10 *w*/*w* are not presented for brevity as they do not exhibit major differences compared to PLA. The main absorption peak of PHSu at 1722 cmcorresponds to the stretching vibration of C=O groups [29,44]. In the area between 1500 and 1380 cm^−1^, the peaks are attributed to methylene (-CH_2_-) scissoring and symmetric deformations. The overlapped peaks in the region 1380–1220 are assigned to waging deformation. The peak at 1155 cm^−1^ is due to stretching vibration of C-O-C groups. Stretching vibration of C-C bonds is between a range of 930 and 610 cm^−1^ [32,45].

The major absorption peak of PLA at approximately 1750 cm^−1^ is attributed to the stretching vibrations of amorphous carbonyl groups [46]. The peak at 1454 cm^−1^ and the double peak at 1383 cm^−1^ and 1358 cm^−1^ correspond to the asymmetric bending of CH_3_ and to the symmetric vibrations of CH_3_ and CH, respectively. The absorption peak at 1179 cm^−1^ is due to the C-O-C stretching vibration, while the shoulder at 1210 cm^−1^ is assigned as the C-O-C stretching characteristic for the crystalline phase. Moreover, the triple band between 1150 and 980 cm^−1^ is also attributed to the stretching mode of C-O-C. Finally, the peak at 868 cm^−1^ is due to absorption of O–CH–CH_3_ and that of 753 cm^−1^ to the wagging absorption of CH_3_ [47].

The spectrum of Risperidone is quite complicated with lots of peaks. The band between 3100 and 2630 cm^−1^ corresponds to the C-H stretching of the aromatic ring [24]. The strong bands consisting of three peaks at 1643 (major), 1610 and 1590 cm^−1^ are attributed to the C=O stretching of the δ-lactam ring and the C=C stretching of the aromatic ring, respectively. The double peak at 1534 cm^−1^ corresponds to the C-N and C-O angular deformations of the oxazole peak. The peak at 1350 cm^−1^ is due to the intermediate band of C-N of the oxazole ring [31]. Wagging of the CH_2_ can be seen at 1412 and 1395 cm^−1^ [48]. Moreover, FTIR-ATR peaks are also observed at 1191 and 1129 cm^−1^, owing to the tertiary amine of the piperidine ring and to the aryl fluoride, respectively [49].

In PLA-b-PHSu80/20 and the other two copolymers, the most evident peaks are those of PLA, because of the small amount of PHSu. The main peak of PLA-b-PHSu80/20 compared to neat PLA is broader and this trend also follows on the prepared microparticles. Comparing PLA-RIS microparticles with neat PLA and PLA-b-PHSu80/20-RIS microparticles with PLA-b-PHSu80/20, it can be seen that the two main peaks of Risperidone are presented in the spectra. The peak at 1533 cm^−1^ is spotted in the same position in both RIS-microparticles, while the main peak at 1643 cm^−1^ is shifted to lower wavenumbers at 1653 cm^−1^.

#### 3.2.3. X-ray Diffraction (XRD)

**Figure 8:**
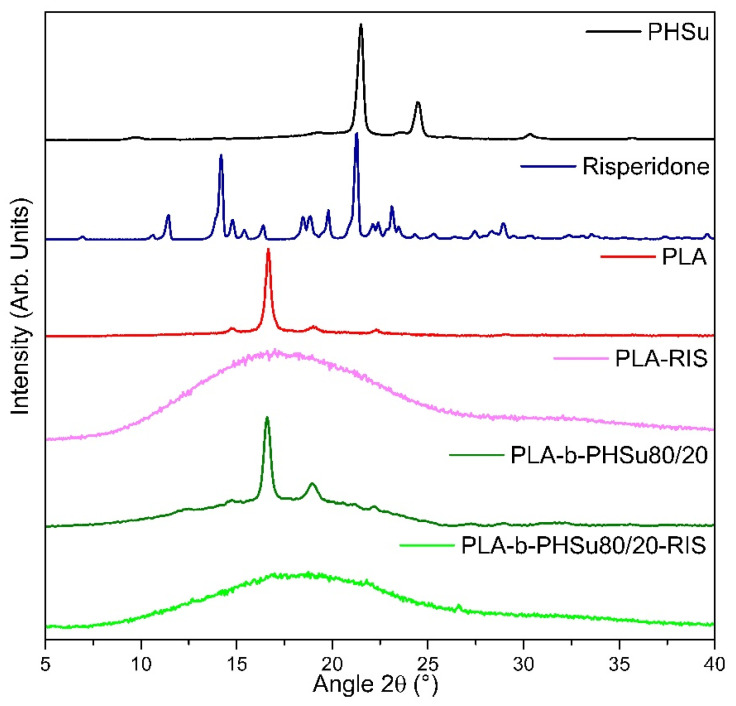
X-ray diffractograms of PHSu, Risperidone, PLA, PLA-b-Phsu80/20 and of the PLA-RIS and PLA-b-Phsu80/20-RIS prepared microparticles.

Figure 8 presents the XRD spectra obtained from PHSu, Risperidone, PLA and PLA-b-PHSu80/20 as well as the micro-PLA and micro-PLA-b-PHSu80/20 after the spay-drying method used to produce RIS microparticles. For brevity, the copolymers in 95/05 *w*/*w* and 90/10 *w*/*w* are not presented. PHSu is a semi-crystalline polymer with the main peaks at 21.4° and 24.4° being attributed to (220) and (040) planes, respectively [44,50]. Risperidone spectra were confirmed by the literature [49,51]. It is a highly crystalline substance with a well-defined X-ray diffraction pattern, with the major reflection at 21.2°. The other characteristic peaks can be spotted at 2θ of 11.4, 14.2, 14.8, 16.4, 19.8, 23.1 and 28.9°.

PLA can be found in amorphous or semi-crystalline state, depending on the thermal processes that it undergoes. In the current study, both PLA and its copolymers after their synthesis by two-stage melt polycondensation [29] and the film processing were in a semi-crystalline state. The main peaks of PLA that are also characteristic in the copolymers at 14.5, 16.6, 19 and 22.3° are assigned to the (010), (110)/(200), (230) and to the (015) planes, respectively. Moreover, it was observed that the copolymers present bigger amorphous phase compared to PLA, meaning that the degree of crystallinity of the copolymers decreases [29].

On the other hand, the XRD spectra of PLA-RIS microparticles showed an amorphous state. The same trend was spotted in each ratio for the microparticles. Thus, it can be assumed that the amorphization of the microparticles and the absence of any Risperidone peak is due to the spray-drying treatment and the homogeneous dispersion of Risperidone within the polymer matrix.

#### 3.2.4. Differential Scanning Calorimetry (DSC)

**Figure 9:**
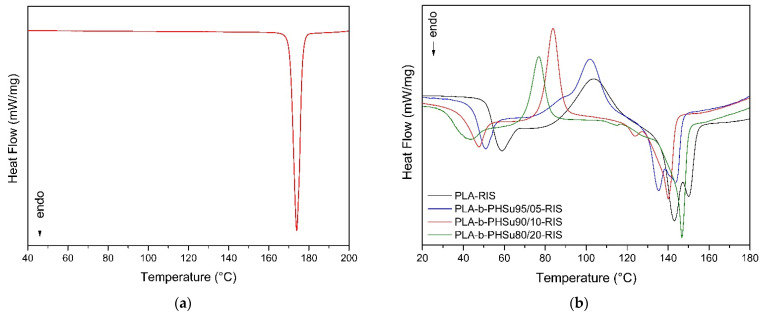
DSC thermograms of RIS (**a**) and the 1st heating of the prepared microparticles (**b**).

Figure 9a presents a DSC thermogram of Risperidone during heating, in which only the melting point of the drug is recorded at 174 °C. The first heating of the prepared microparticles (Figure 9b) represents the heating of the samples as received after spray drying and also used for the XRD measurements. The first thing that is observed is the existence of a crystallization peak, meaning amorphous samples are in total agreement with the XRD results. Moreover, the glass transition temperature can be clearly seen in every sample and is shifted to lower temperatures by increasing the PHSu content. The exothermic peaks that are following correspond to the crystallization of the samples. As it is observed, the peaks of micro PLA and those of the 95/05 *w*/*w* sample are wider and slightly overlap with the melting peaks. On the other hand, those of 90/10 and 80/20 *w*/*w* are sharper and distinct from the melting peaks. Concerning the endothermic peaks, they consist of a double melting peak characteristic of PLA. Micro PLA-b-PHSu90/10 and PLA-b-PHSu80/20 have one main melting peak, while a small endothermic peak appears as well. The existence of two melting peaks and the Tg was also seen in the initial samples without Risperidone before spay drying [29]. The melting point of Risperidone drug was not recorded, indicating that the drug was probably encapsulated in its amorphous form, assuming the formation of a solid dispersion of RIS in the copolymer during the solvent evaporation process. This is in good agreement with XRD studies. Additionally, the absence of the Risperidone peak could also be explained by its dissolution into the polymer matrix, which became liquid during heating [52].

#### 3.2.5. Thermogravimetric Analysis (TGA)

**Figure 10:**
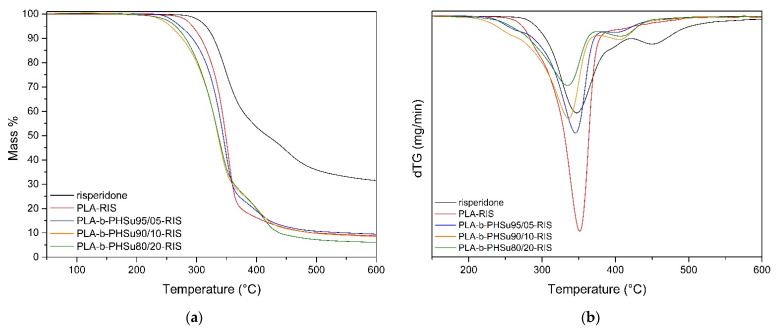
Mass (%) (**a**) and dTG (**b**) versus temperature curves obtained from TGA experiments.

The thermograms of Risperidone and the microparticles are presented in Figure 10. From the mass loss versus temperature diagram (Figure 10a), it can be seen that Risperidone has approximately 30% remaining mass at 600 °C, while it consists of two degradation steps. The first step occurs in a temperature range from 262 °C to 421 °C and presents a decrease of 50.3%, while the second from 421 °C to 540 °C has a 15.9% decrease in mass. As for micro-PLA and the micro-PLA-b-PHSu copolymers, they present the same thermal behavior with the initial bulk polymers, as already thoroughly discussed in our previous article [3]. It can be observed that the microparticles present a slight remaining mass of 8% (micro PLA, PLA-b-PHSu 95/05 and PLA-b-PHSu 90/10 *w*/*w*) and 6% on the PLA-b-PHSu 80/20 *w*/*w* sample, which corresponds to the small amount of Risperidone. From this study, it is clear that the Risperidone encapsulation has no effect on the thermal stability behavior of microspheres.

#### 3.2.6. In Vitro Drug Release

Table 1 shows the drug loading and entrapment efficiency of Risperidone in microparticles. At this point, it must be mentioned that the yield of the microparticles was not calculated since no trap for smaller microparticles was adapted to the exit of the cyclone. Further, it is known that about 20% of the inlet mass is lost on the vessel’s wall. Initial drug load in the solution was 14.53%. After spray drying, drug loading was found to be 10.61% for PLA microparticles, showing a slight increase compared to the drug loading obtained by single emulsification (o/w) in a previous study [24]. Hence, the resulting microspheres in this study are smaller, about 10 μm in size, and their drug loading is considered to be high enough. The drug loading for microparticles prepared with PLA-b-PHSu95/5 copolymer was analogous (10.77%), while as the content of PHSu increased, the drug loading was decreased to 9.52% and 8.43%, respectively. This is probably attributed to the increasing hexylene succinate content; as the content of hexylene succinate increased, the acidic polymer ratio was decreased, leading to lower neutralization of Risperidone and to lower drug loading values [53]. Analogous are the results found for %EE values. Entrapment efficiency (EE) for PLA was found to be 73.16%, an enhanced value compared to the one from typical o/w procedure (36.51%). This was expected since the microparticles formed during the spray-drying procedure are not subjected to other processes, such as overnight stirring in aqueous solution, centrifugation and washing steps, which result in lower entrapment efficiency values. As the content of PHSu in the copolymers increased, the EE decreased, as also previously found for drug loading values.

**Figure 11:**
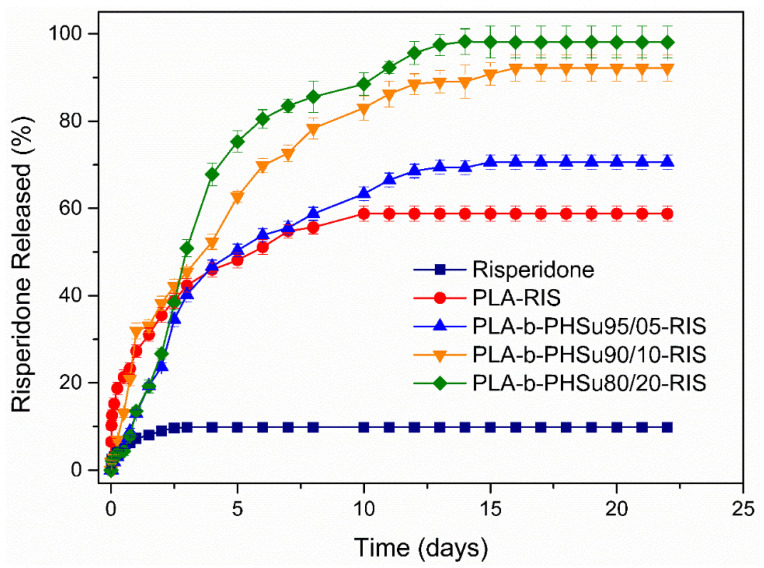
Dissolution rate of Risperidone from prepared microparticles.

The dissolution study of Risperidone from the prepared microparticles is presented in Figure 11. As can be seen, Risperidone dissolves at 10% in PBS in the first 2 days without any further dissolution [24], which can be attributed to the high crystalline phase and the hydrophobicity in the API. All the prepared microparticles enhanced the dissolution profile of Risperidone. In brief, PLA exhibited a release of the drug in two different stages; a first stage, with an initial burst release lasting 1 day (release of 20% of RIS) and a second stage, with a controlled release, reaching about 55% and lasting 10 days, while, thereafter, no release was observed. A similar behavior was observed in our previous study [24] where microparticles of PLA containing RIS were prepared according to the single emulsification method (o/w). In the previous study, it was observed that Risperidone was also dispersed in amorphous state in microparticles, as also found in this study, and the drug was released in a controlled manner, reaching about 50% in 6 days. Hence, microparticles prepared in the previous study were bigger in size (about 17 μm) and their surface was smooth without showing any pore formation. It is known that release of the drug from polymeric matrices is affected by several factors, such as particle size, molecular weight of polymer used, degree of crystallinity and melting point of polymeric matrix [54]. In this study, for all microparticles, it was found that the drug was dispersed in amorphous state and the polymers were also amorphous. Drug amorphization can explain the high dissolution rate from microparticles, compared with the neat drug.

In this work, the initial burst effect observed with PLA microparticles can be attributed to pore formation on the microparticles’ surface or to Risperidone being located mainly close to the microspheres surface, while the controlled release is mainly attributed to release due to diffusion of the drug between the channels formed. The observations for the microparticles prepared with the synthesized copolymers are analogous. In all profiles, an initial burst effect is observed, lasting for the first day and reaching a percentage of 20–30% depending on the ratio of PHSu in the copolymers; as PHSu ratio increased, the percentage of initial burst effect increased as well. In all formulations, burst effect was attributed to the drug present in the microparticles’ surface.

After the first day, microparticles showed differences in Risperidone’s release. In brief, those prepared by PLA-b-PHSu95/05 exhibited an analogous trend to PLA microparticles, showing a controlled release for 12 days, reaching about 68% release, a slight increase to 70% until 14 days without any further change thereafter. Microparticles, having as matrix PLA-b-PHSu90/10 and 80/20, showed enhanced release profiles reaching 90 and 100% release in 15 and 12 days, respectively. This remarkable increase is probably attributed to the kind of microparticles prepared. It seems that in these two cases, poly-nuclear microcapsules were formed [36] since, as can be observed in SEM photos, internal pores are formed in the microparticles. Due to internal pores, diffusion of RIS is easier, resulting in a higher release.

**Figure 12:**
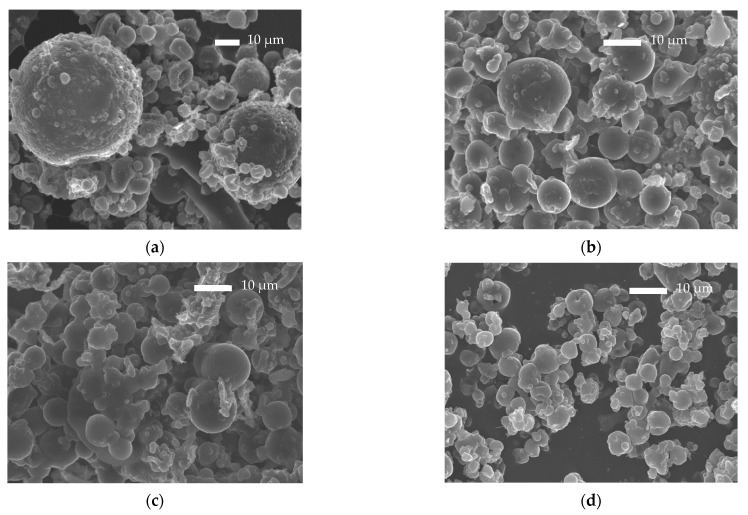
SEM photos of microparticles after release study in 22 days at 37 °C in 100/0 (**a**), 95/05 (**b**), 90/10 (**c**), 80/20 (**d**) ratios.

SEM micrographs were also taken after RIS release, at 22 days. The micrographs are presented in Figure 12. PLA microparticles retained their shapes and sizes after the dissolution study, but a higher agglomeration of smaller microparticles is observed. For PLA-b-PHSu95/05 microparticles, it seems that after drug release, agglomeration also increased without any change in the particle size. Larger deformations in microparticles’ morphology were observed in the other two formulations where the ratio of PHSu was higher. Briefly, the size of microparticles decreased, while most of them seem to lose their spherical shape and important agglomeration is also observed. These alterations can probably be attributed to PHSu blocks that are hydrolyzed more easily.

#### 3.2.7. Analysis and Discussion of Release Results by Mechanistic Release Model

An attempt will be made here to quantitatively describe the drug release kinetics. The classical models used for drug release cannot appropriately represent the data so more advanced models have to be employed. The first step is to consider the release of the pure drug. The drug is dissolved in two steps: (i) transformation of the solid drug to dissolved one and (ii) mass transfer of the dissolved drug from interfacial to liquid bulk area. The kinetics of the second step is promoted through agitation. Under the present conditions, one could argue that the release process is fully dominated by step (i). This step can be formulated in terms of an apparent reaction, whereas for most of the drugs, this reaction is of first order [55,56]; this is not the case for Risperidone. General *n*-th order reaction kinetics is considered. Denoting as X the ratio between actual drug concentration C and solubility C_eq_, the reaction kinetic model can be written as (where *K* is the kinetic constant):

(5) $$\frac{dX}{dt}=-K{\left(1-X\right)}^{n}$$

Integrating, using *X*(0) = 0 leads to

(6) $$X=1-{\left(1+\left(n-1\right)Kt\right)}^{1/\left(1-n\right)}$$

**Figure 13:**
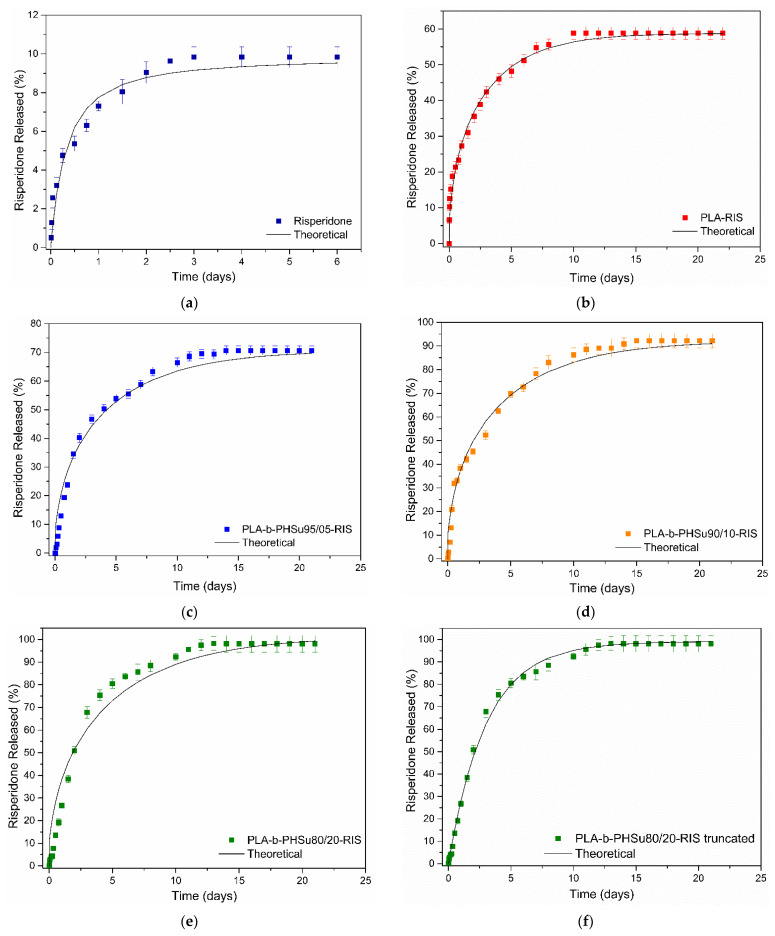
Comparison between experimental and theoretical drug release curves (**a**) Risperidone API-Equation (6); (**b**) PLA-Equation (7); (**c**) PLA-b-PHSu95/5-Equation (7); (**d**) PLA-b-PHSu90/10-Equation (7); (**e**) PLA-b-PHSu80/20-Equation (7); (**f**) PLA-b-PHSu80/20-Equation (8).

The *X* evolution curve can be transformed to a release curve of the type appearing in Figure 10, through a multiplication with *X_f_* = 100 C_eq_V/m where V is the liquid volume and m is the total drug mass. A fitting procedure of the model to the data leads to the unknown parameter values. The fitting parameters are *X_f_* = 9.8, *K* = 3.52·10^−5^ s^−1^, *n* = 1.78. The comparison between data and fitted model is presented in Figure 13a.

The release from polymeric particles undergoes more complex kinetics. The driving force is the combination of a diffusional mechanism with an erosional one. Additional complexities are coming from the spatial distribution of the drug in the particles, from the shape of the particles and from the particle size distribution. The problem is underdetermined in general, so it is not possible to extract the spatial drug distribution based only on release curves. However, specific assumptions allow the derivation of realistic kinetic models supporting the analysis of the data. The main assumptions invoked here are the simultaneous action of polymer hydrolysis and of Fickian drug diffusion. The spatial distribution of the drug in the particles is assumed to be uniform. In addition, a characteristic spherical shape of the particles is considered having a representative radius equal to R. The extent of the hydrolysis is, in general, small, according to the experiments shown in Figure 2. In such a situation, the release due to erosion and the release due to diffusion can be assumed to proceed independently. The release curve for such a situation has the form (including the initial burst effect) [57]:

(7) $$X=X_{f}\left(1-\frac{6}{\pi^{2}}\overset{\infty }{\underset{i=1}{\sum }}\frac{1}{i^{2}}\exp\left(-ki^{2}\pi^{2}t\right)\right)+H\left(t\right)$$

where *X* is the fraction of the drug released at time *t*, *X_f_* is the equilibrium release due to diffusion, *H*(*t*) is the contribution of hydrolysis to release and *k* is a kinetic constant related to the diffusion coefficient of drug in polymer matrix D and to particle size R as *k* = D/R^2^. The function *H*(*t*) is very small for pure PLA and PLA-b-PHSu95/05 so it is ignored. An exponential of the form *H*(*t*) = *H_f_*(1 − exp(−α*t*)) was used to fit the hydrolysis data for the 90/10 *w*/*w* (*H_f_* = 1.45, α = 6.9·10^−7^ s^−1^) and for 80/20 *w*/*w* particles (*H_f_* = 3.35, α = 1.38·10^−6^ s^−1^).

The next step is to fit Equation (7) to the experimental release data by proper selection of the parameters *X_f_* and *k*. The fitting parameters appear in Table 2. The quality of the fit appears in Figure 13b–e. It is noted that the theoretical curves closely reproduce the initial burst effect, except in 80/20 particle case. This suggests that the drug distribution in the three cases is a uniform one and that the initial release burst observed is due to the structure of the diffusion equation. The situation is quite different for the 80/20 particles. It is obvious that the experimental data do not follow the theoretical release burst (Figure 13e). This suggests the possibility of the initial lack of the drug at the outer region of the particle. A simplified solution of the diffusion equation known as Linear Driving Force formula can be invoked in that case [58]. According to this approximation, the release evolution is given as

(8) $$X=X_{f}\left(1-\exp\left(-Kt\right)\right)+H\left(t\right)$$

where *K* = 15D/(R^2^). The 80/20 particle release data are fitted using the above formula and as it appears in Figure 13f, the quality of fitting is high. This confirms that in the case of 80/20 particles, there is a lack of drug at the outer region of the particles. The parameter *k* appears to decrease as the fraction of PHSu in the particle increases. Considering a representative particle radius of R = 5 μm, the drug diffusivity values appear to be of the order of 5·10^−18^ m^2^/s. The coefficient K in Equation (8) used to fit the data is 3.93·10^−6^ s^−1^. The diffusivity in this case is computed as 6.5·10^−18^ m^2^/s, revealing the consistency of the approach (similar diffusivities from different approximations). Summarizing, it appears that as PHSu fraction in the polymer increases, the effect of erosion increases and the diffusivity of the drug in the particle decreases. The spatial distribution of the drug in the particle is uniform except for the PLA-b-PHSu80/20 particles, for which there is a lack of drug at the outer part of the particles.

## 4. Conclusions

The aim of this work was to study the impact of the incorporation of PHSu blocks on the biodegradation of PLA and the preparation of PLA/PHSu microparticles, where Risperidone was incorporated for drug delivery systems. Initially, NMR in conjunction with SEC measurements previously published confirmed the block structure of the copolymers. Enzymatic hydrolysis showed a decrease in weight loss as the content of PHSu increased, while SEM microphotographs confirmed the results, i.e., increased surface roughness with increasing PHSu content. The cytotoxicity study confirmed the biocompatibility of the copolymers. As for the microparticles, their spherical shape was confirmed by SEM and Risperidone incorporation, verified by FTIR. As the content of PHSu in the copolymers increased, the drug loading and the entrapment efficiency decreased. On the other hand, concerning the release of Risperidone, as the content of PHSu increased, a higher percentage of RIS was released, with PLA-b-PHSu80/20 releasing 100% of RIS after 12 days. From the drug release kinetic model, it was found that the RIS release is controlled by a complex mechanism of erosion and diffusion. As PHSu fraction in the polymer increases, the effect of erosion increases too, while drug diffusion in the particle decreases.

## Figures and Tables

**Table 1:** Drug loading content and %EE of RIS prepared microspheres.

Sample	Drug Loading (%)	Entrapment Efficiency (%)
micro PLA	10.61 ± 1.32	73.16 ± 2.09
micro PLA-b-PHSu95/5	10.77 ± 1.05	74.26 ± 2.11
micro PLA-b-PHSu90/10	9.52 ± 0.98	65.63 ± 1.67
micro PLA-b-PHSu80/20	8.43 ± 0.95	58.15 ± 1.48

**Table 2:** Values of parameters resulting from fitting Equation (7) to experimental release curves.

Sample	*X_f_*	*k* (s^−1^)
micro PLA	58.8	3.18·10^−7^
micro PLA-b-PHSu95/5	70.6	2.12·10^−7^
micro PLA-b-PHSu90/10	90.8	2.15·10^−7^
micro PLA-b-PHSu80/20	97.8	1.95·10^−7^

## Data Availability

The data presented in this study are available on request from the corresponding author.

## Supplementary Materials

The following supporting information can be downloaded at: https://www.mdpi.com/article/10.3390/polym14194111/s1, Figure S1: SEM microphotographs of PLA (a,b), PLA-b-PHSu 95/05 *w*/*w* (c,d), PLA-b-PHSu 90/10 *w*/*w* (e,f) and PLA-b-PHSu 80/20 *w*/*w* (g,h) before and after hydrolysis respectively.
