# Supplementary material for: Poly(Lactic Acid) Block Copolymers with Poly(Hexylene Succinate) as Microparticles for Long-Acting Injectables of Risperidone Drug
Source: Polymers (Basel). 2022 Sep 30;14(19):4111. doi: 10.3390/polym14194111 (PMC9571843; doi:10.3390/polym14194111)
Supplement: Supplementary file 1 [file polymers-14-04111-s001.zip › polymers-1907971-supplementary.pdf]

polymers

MDPI

Supplementary Materials

Poly(Lactic Acid) Block Copolymers with Poly(Hexylene Succinate) as Microparticles for Long-Acting Injectables of Risperidone Drug

Iouliana Chrysafi [1], Stavroula Nanaki [2], Alexandra Zamboulis [2], Margaritis Kostoglou [3], Eleni Pavlidou [1] and Dimitrios N. Bikiaris [2,*]

1 Laboratory of Advanced Materials and Devices, Department of Physics, Faculty of Sciences, Aristotle University of Thessaloniki, GR-541 24 Thessaloniki, Greece
2 Laboratory of Polymers Chemistry and Technology, Department of Chemistry, Faculty of Sciences, Aristotle University of Thessaloniki, GR-541 24 Thessaloniki, Greece
3 Laboratory of Chemical and Environmental Technology, Aristotle University of Thessaloniki, GR-541 24 Thessaloniki, Greece
* Correspondence: dbic@chem.auth.gr

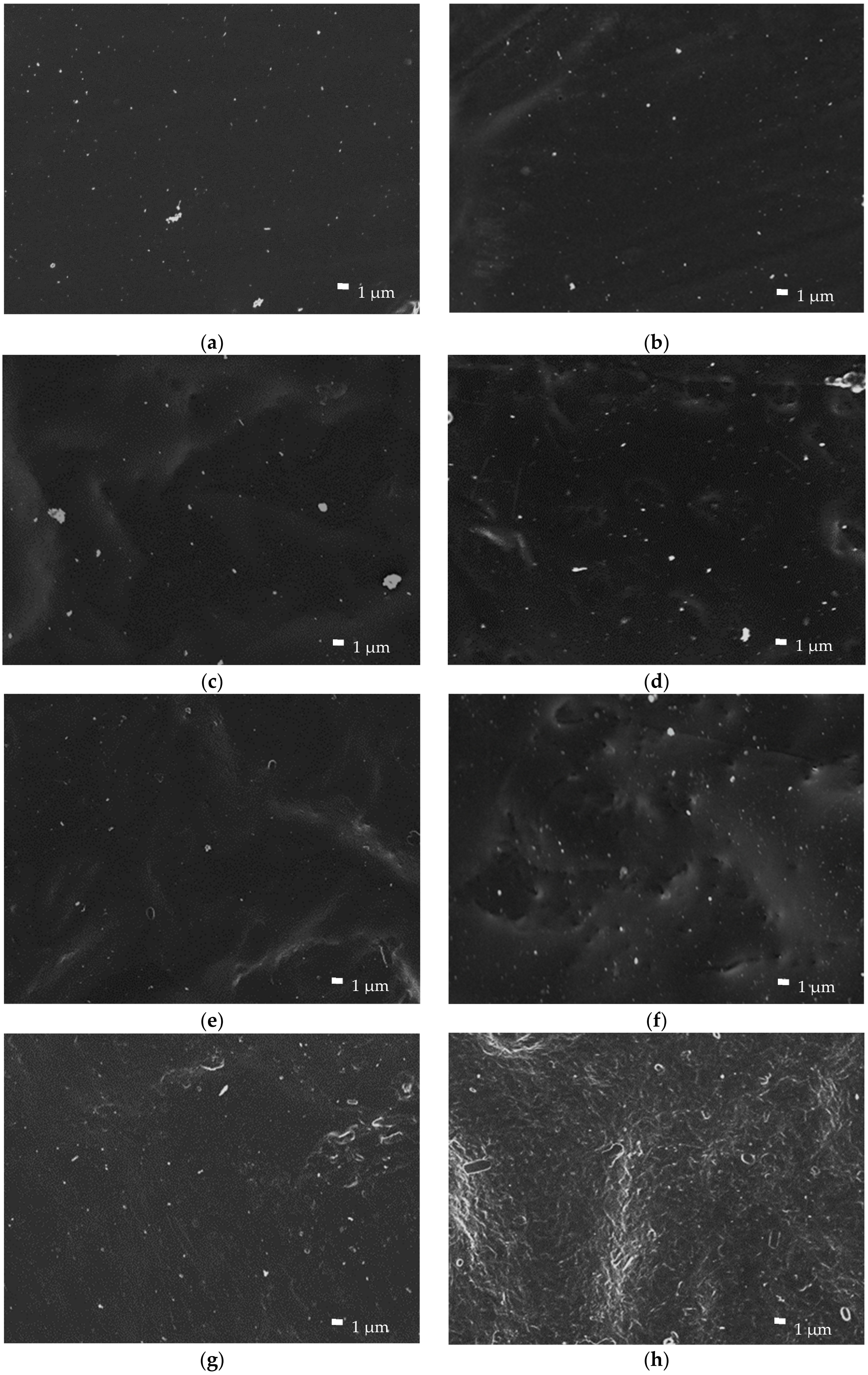

Figure S1. SEM microphotographs of PLA (a,b), PLA-b-PHSu 95/05 w/w (c,d), PLA-b-PHSu 90/10 w/w (e,f) and PLA-b-PHSu 80/20 w/w (g,h) before and after hydrolysis respectively.
